# Radix Asteris: Traditional Usage, Phytochemistry and Pharmacology of An Important Traditional Chinese Medicine

**DOI:** 10.3390/molecules27175388

**Published:** 2022-08-24

**Authors:** Ke-Jie Li, Yang-Yang Liu, Dong Wang, Pei-Zheng Yan, De-Chao Lu, Dong-Sheng Zhao

**Affiliations:** 1 Experimental Center, Shandong University of Traditional Chinese Medicine, Jinan 250355, China; 2 College of Pharmacy, Shandong University of Traditional Chinese Medicine, Jinan 250355, China; 3 Shandong Academy of Pharmaceutical Sciences, Jinan 250101, China; 4 International Education College, Shandong University of Traditional Chinese Medicine, Jinan 250355, China

**Keywords:** *Aster tataricus* L. f., traditional uses, chemical constituents, pharmacology, quality control

## Abstract

Radix Asteris (RA), also known as ‘Zi Wan’, is the dried root and rhizome of *Aster tataricus* L. f., which has been used to treat cough and asthma in many countries such as China, Japan, Korea and Vietnam. This article summarizes the available information on RA in ancient Chinese medicine books and modern research literature: its botanical properties, traditional uses, chemical composition, pharmacological activity, toxicity and quality control. Studies have shown that RA extracts contain terpenes, triterpenoid saponins, organic acids, peptides and flavonoids, and have various pharmacological activities such as anti-inflammatory, anti-tumor, anti-oxidation, and anti-depression. RA is considered to be a promising medicinal plant based on its traditional use, chemical constituents and pharmacological activities. However, there are few studies on its toxicity and the consistency of its components, which indicates the need for further in-depth studies on the toxicity and quality control of RA and its extracts.

## 1. Introduction

Radix Asteris (RA) is the dried root and rhizome of *Aster tataricus* L. f. belonging to the Asteraceae family [1]. It is widely distributed in the low mountain shady slope wetlands, mountain tops and low mountain grasslands and swamps in the northeast and northwest of China, North Korea, Japan and eastern Siberia, Russia [2].

RA was first recorded in Shen Nong’s Materia Medica (Shen-Nong-Ben-Cao-Jing) as having effects of moistening the lungs and lowering the qi, eliminating phlegm and relieving cough in the treatment of diseases such as cough and asthma [3,4]. Modern pharmacological studies show that RA extract can effectively reduce the frequency of coughs induced by ammonia in mice [5]. Saponins and 4-hydroxyphenylacetic acids isolated from RA have the potential to treat acute lung injury [6,7]. Shionone, as a marker and a content determination index for RA quality control in the Chinese Pharmacopoeia (2020), is considered to be an active ingredient in RA extract for its expectorant and antitussive activities in mouse models [1]. In addition, RA extract has also been reported to have anti-tumor, antibacterial, antioxidant and other activities [8].

Although much relevant literature has been published on its chemical constituents and biological activities, there is no systematic summary of the body of scientific information describing RA with an emphasis on its medicinal value. Therefore, this study systematically reviews the botany, ethnopharmacology, chemical composition, pharmacological activities, toxicity and quality control related to RA. The aim is to provide a valuable comprehensive reference for the further development and utilization of this important natural medicinal resource.

## 2. Methodology

In September 2021, our team began to collect information on RA, including from Web of Science, CNKI, China Duxiu Scholar, Wanfang Data Platform, PubMed, Google Scholar, SciFinder Scholar, Springer, and Baidu Scholar, as well as ancient Chinese Materia Medica writings. Considering language and text limitations, this article only utilizes Chinese and English texts. Searches were carried out for RA using a combination of keywords, including: “Radix Asteris”, “*Aster tataricus* L. f.”, “Aster” and “Aster genus”, “Pharmacological Activity”, “Chemical Ingredients”, “Toxicity” and “Quality Control”; literature on protection and plant cultivation excluded. The scientific names and photos of RA were obtained from *Flora of China* [2]. In January 2022, the traditional usage of RA began to be summarized and the chemical compositions were produced by ChemDraw. In March 2022, pharmacology, toxicology knowledge and quality control began to be summarized, and a legend was drawn.

## 3. Botany and Ethnopharmacology

### 3.1. Botany

**Figure 1:**
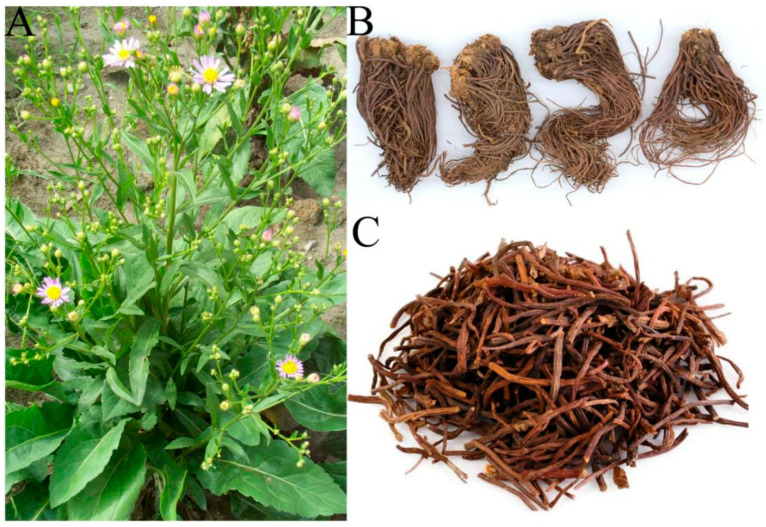
The aerial parts (**A**), roots (**B**), and TCM decoction pieces (**C**) of Radix Asteris.

RA (Figure 1) is a perennial herb with a sloping rhizome. Stems, about 40–50 cm high, are erect and stout, with fibrous dead leaf fragments at the base. Plants are sparsely shaggy, with sparse leaves and ribbed and furrowed adventitious roots. There can be many flower heads, 2.5–4.5 cm in diameter, arranged in compound corymbs at the stem and branch ends. Often there can be about 20 ligulate flowers, tube length 3 mm, tongue blue-purple, 15–17 mm long, 2.5–3.5 mm wide with many veins [2].

### 3.2. Ethnopharmacology

RA was first recorded in the ancient medical work *Shen Nong Ben Cao Jing* (Han Dynasty), and subsequently written about in many ancient herbal works, such as the *Wu Pu Materia Medica* (Wei Jin, 420–589 AD), the *Ben Cao Jing Shu* (Ming, 1625 AD), and the *Ben Cao Feng Yuan* (Qing, 1695 AD) for its multiple effects of “warming” the lungs, relieving coughs, eliminating phlegm and lowering qi. The records of RA in ancient Materia Medica are listed in Table 1.

RA were excavated in spring and autumn, and braided and dried in the sun, or directly dried after removal of the knotted rhizomes (commonly known as “mother roots”) and sediment. It is called “Sheng Zi Wan” when directly dried and “Mi Zi Wan” when processed with refined honey [9]. In 1963, RA was included in the Pharmacopoeia of the People’s Republic of China. In most cases, RA is used in combination with other TCMs to form prescriptions for the treatment of wind-cold coughs, asthma, consumptive coughs, vomiting, puss formation and bleeding. Ten representative formulations containing RA are listed in Table 2. 

## 4. Chemical Composition

To date, 135 compounds (Table 3) have been isolated from RA, mainly including terpenes, organic acids, peptides, flavonoids and other compounds. These are listed in Table 3.

### 4.1. Terpenes

**Figure 2:**
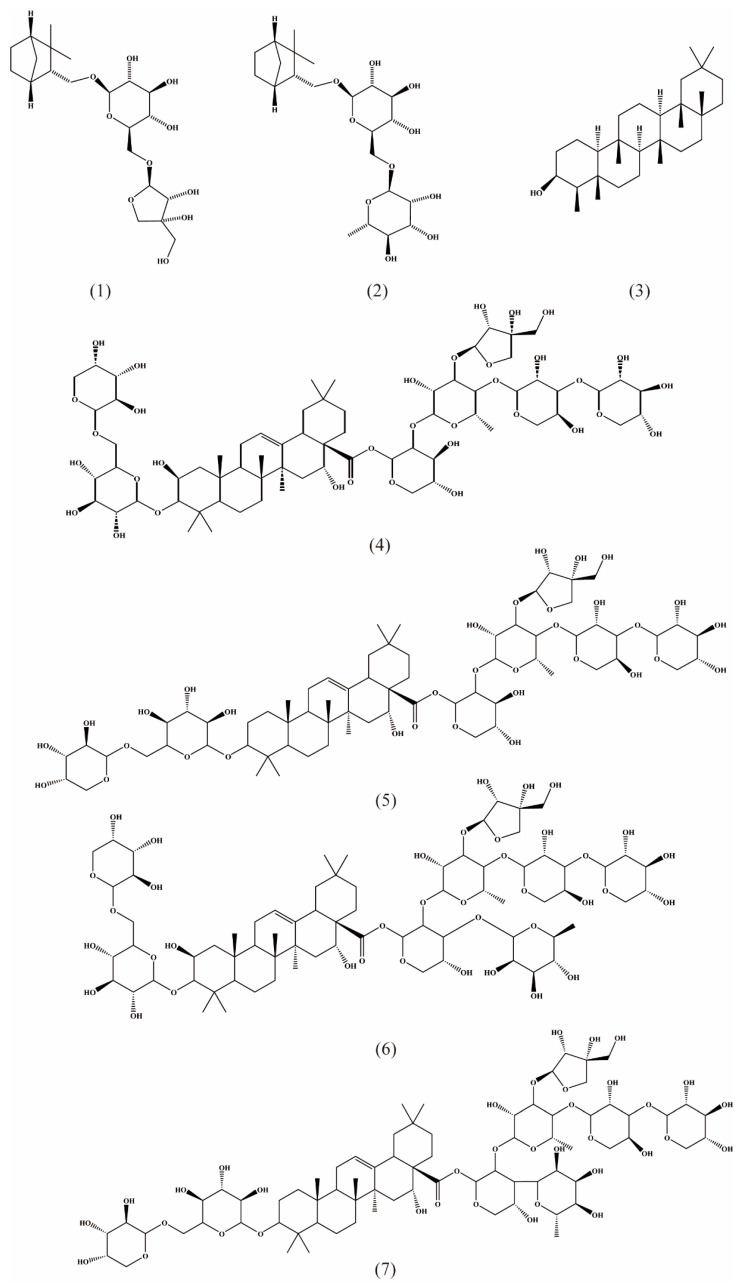

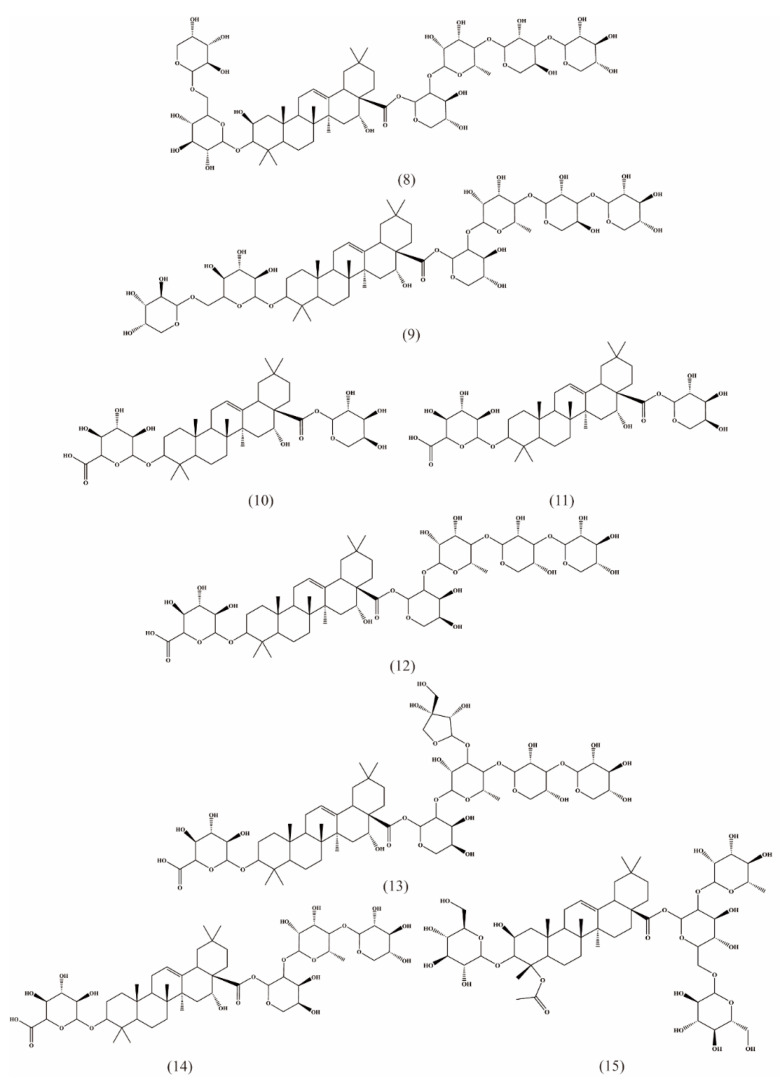

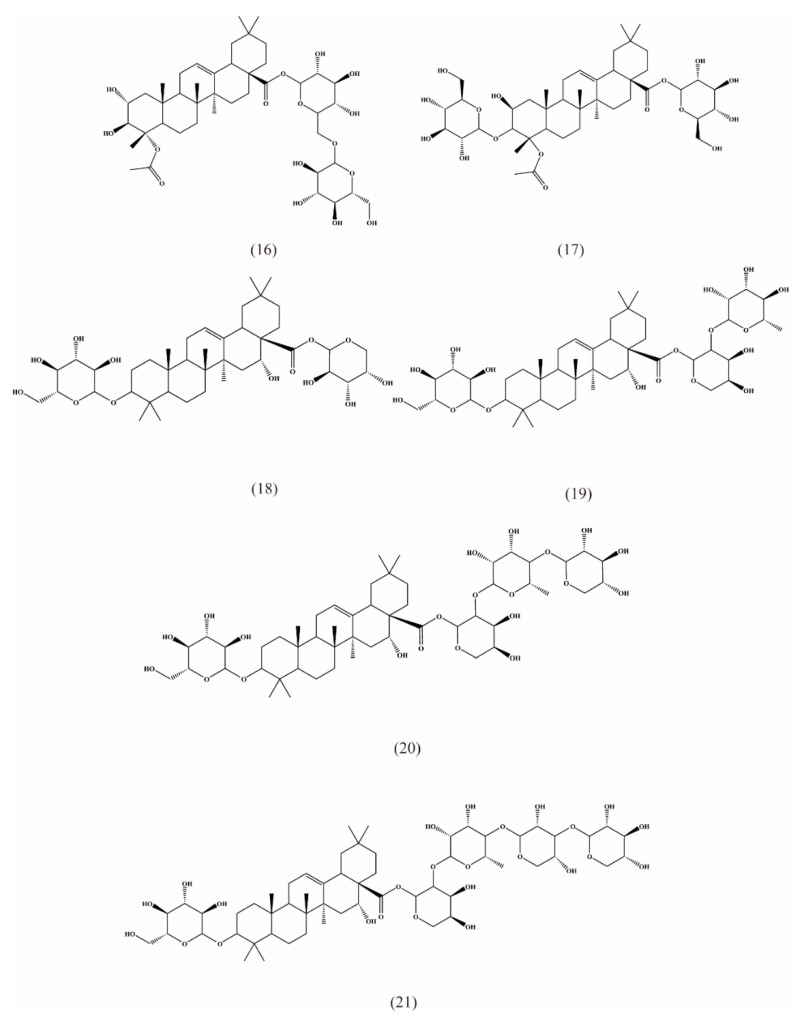

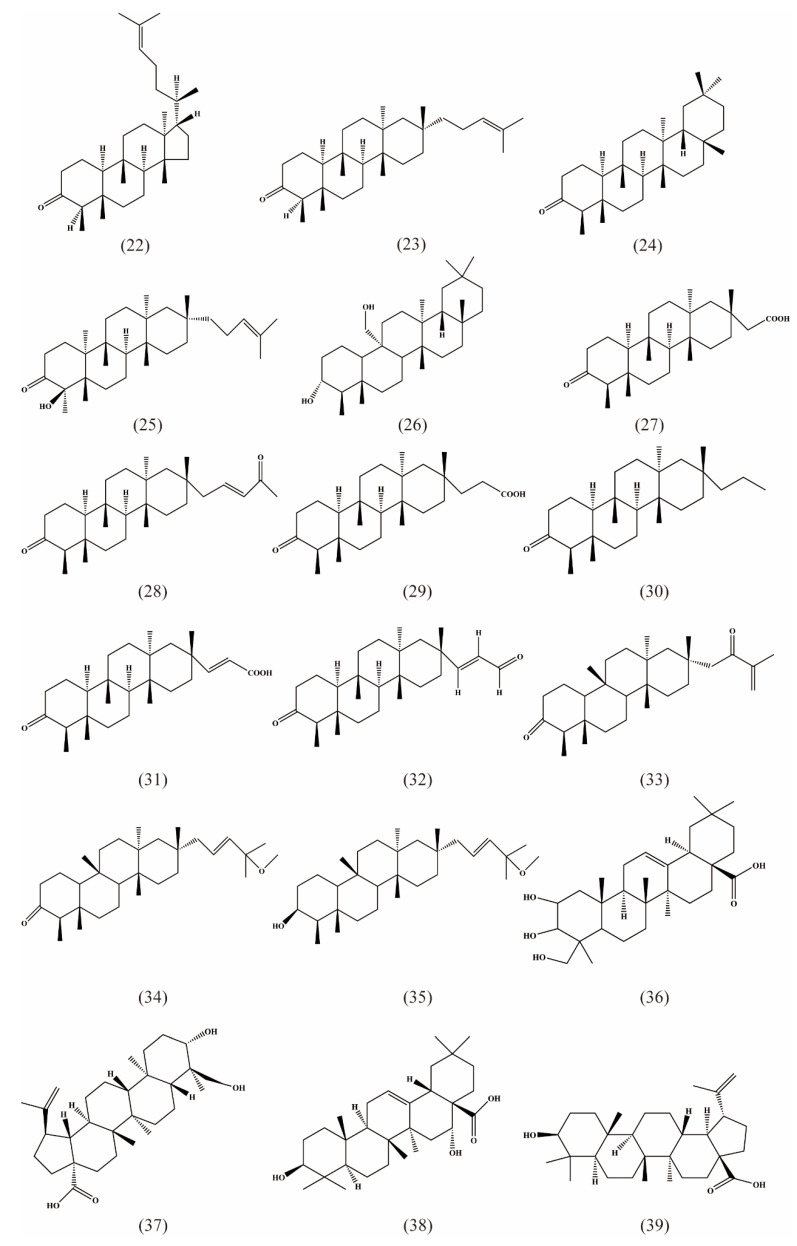

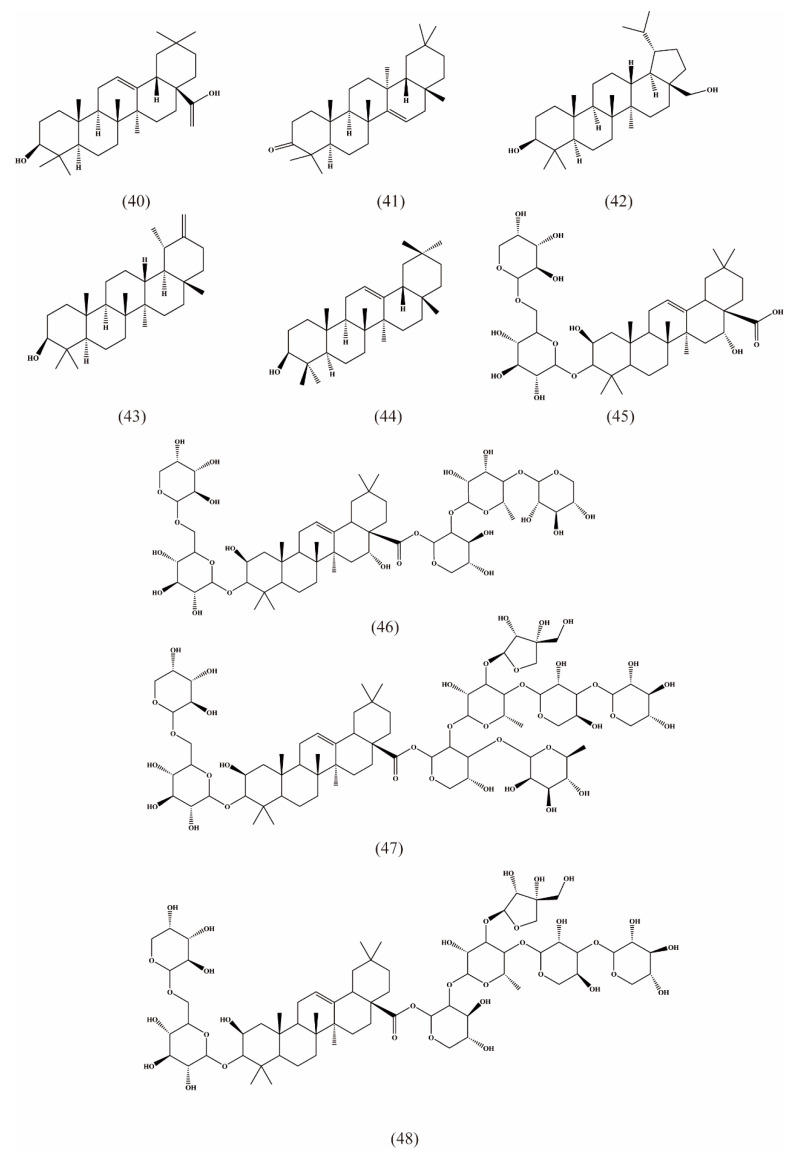

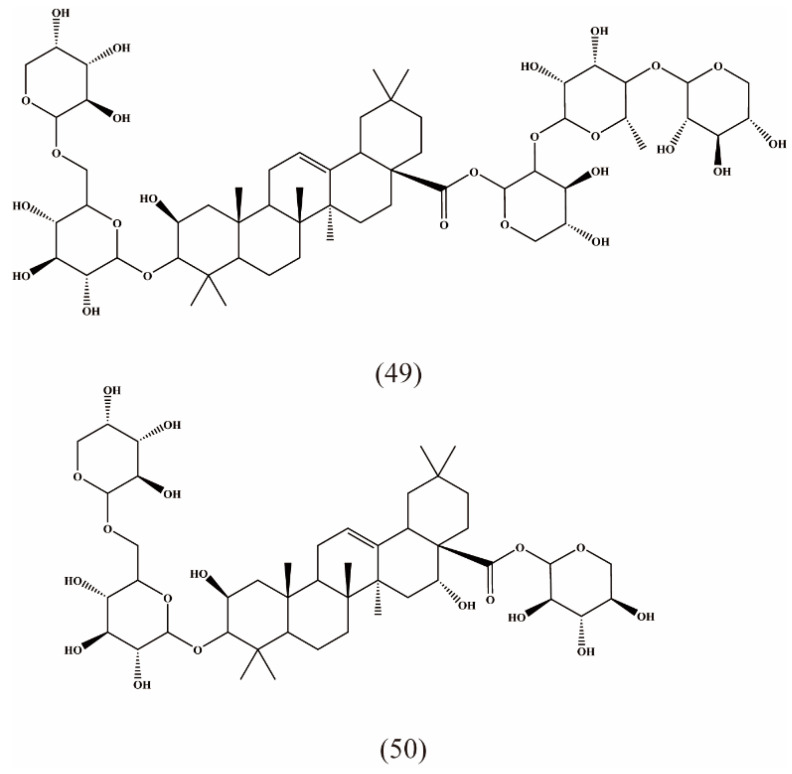
Structures of terpenes isolated from Radix Asteris.

Terpenoids are the most abundant class of compounds in RA, including triterpenoids (Table 3, Figure 2 (3,22–44)), mono-glycosides (Table 3, Figure 2 (1,2)), and triterpenoid saponins (Table 3, Figure 2 (4–21,45–50)), with a total of 50 identified from its different parts, in which there are five from the aerial parts (Table 3, Figure 2 (10–14)), four from the whole plant (Table 3, Figure 2 (18–21)) and 41 from the underground parts (roots and rhizomes). Triterpenoid saponins are one of the important active ingredients [31] and the main ingredient with an expectorant effect [32]. Shionone, as a specific triterpene, has been used as a marker compound for quality control of RA in the Chinese Pharmacopoeia [1].

### 4.2. Organic Acids

**Figure 3:**
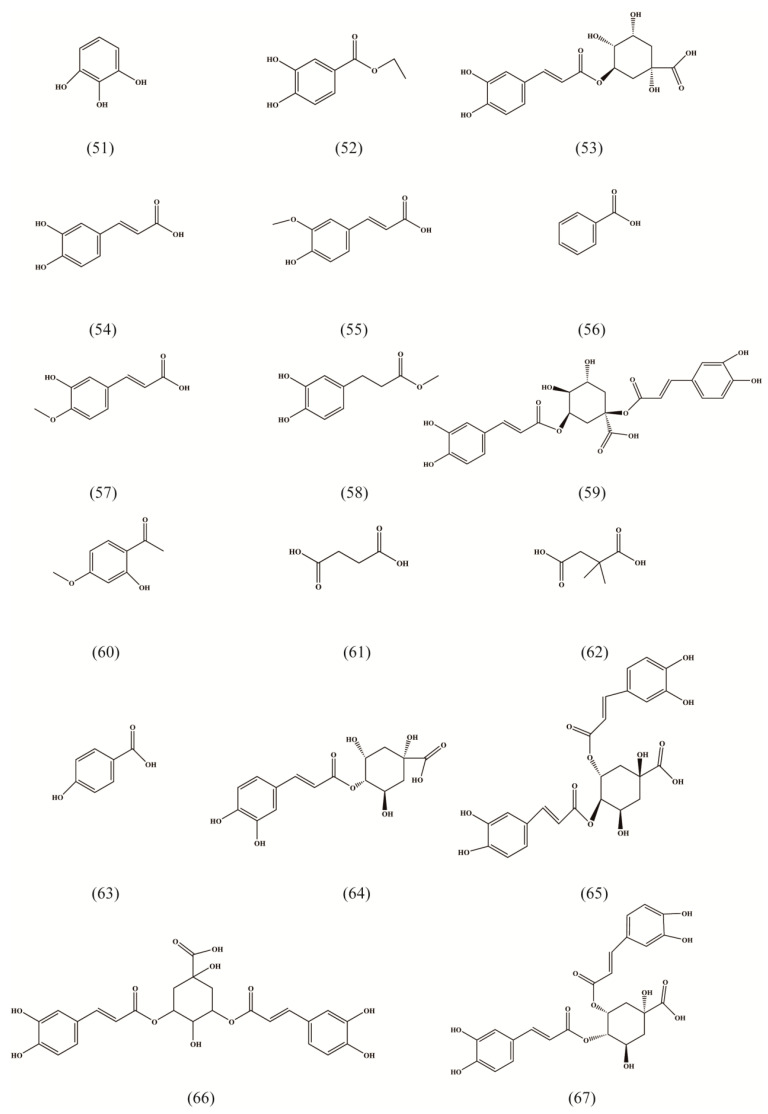
Structures of organic acids isolated from Radix Asteris.

Organic acids are an important class of compounds in RA, and play an important role in anti-stress, anti-thrombosis and anti-inflammatory treatments [30]. Up to now, a total of 19 organic acids, mainly aromatic organic acids and only two saturated chain organic acids, have been found in the root and rhizome of RA (Table 3, Figure 3 (61–62)) [23,33].

### 4.3. Peptides

**Figure 4:**
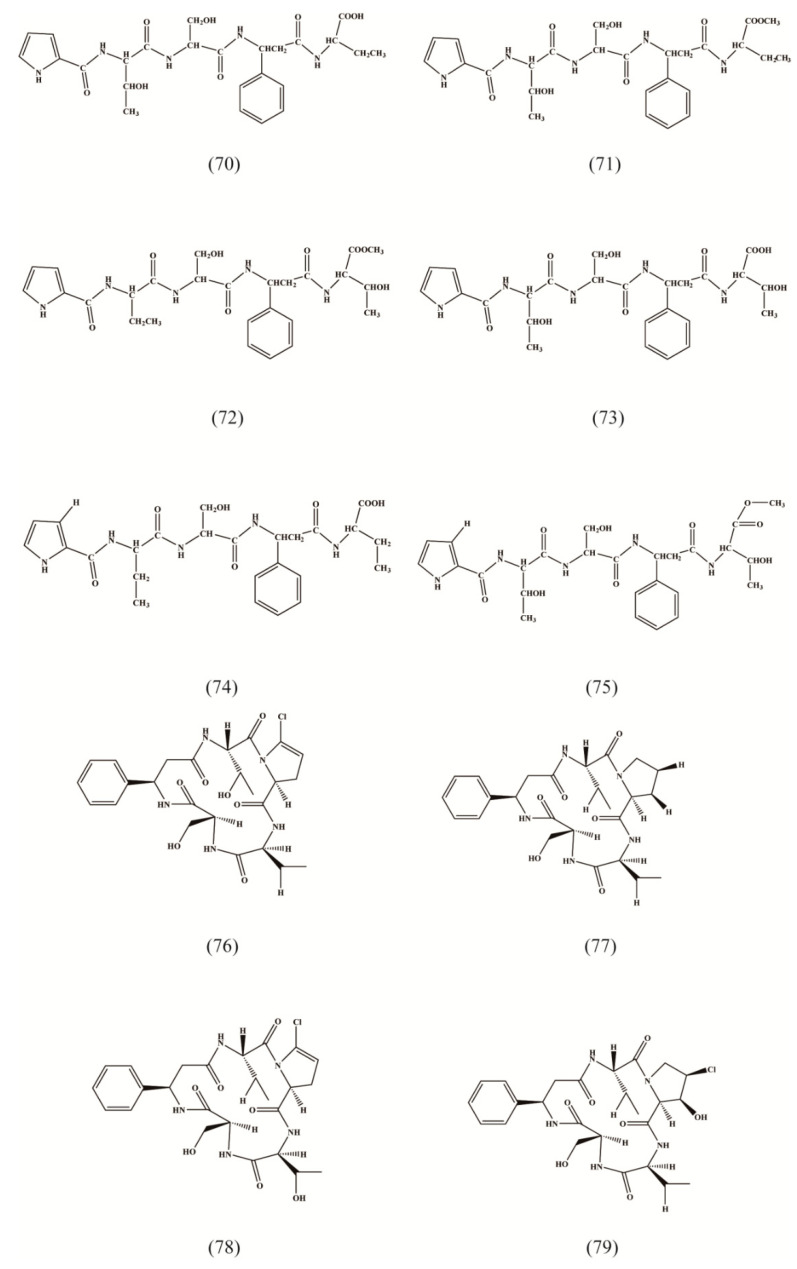

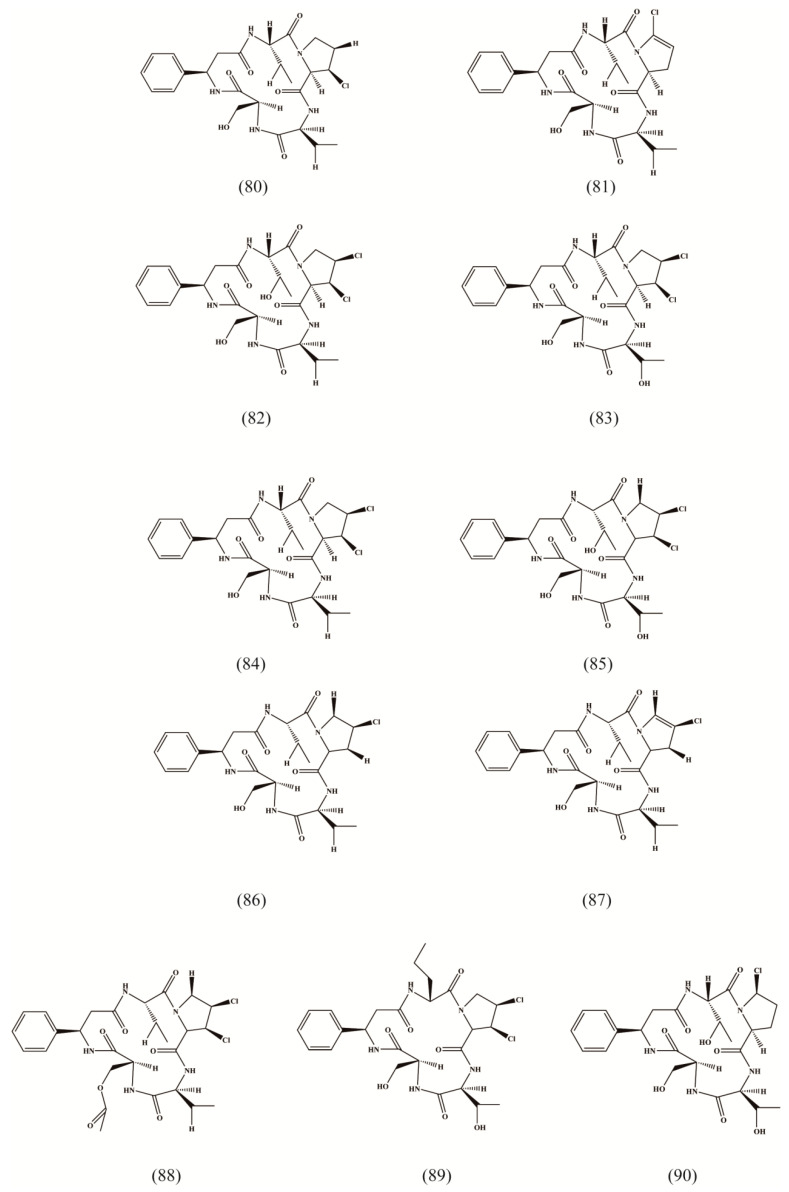
Structures of peptides isolated from Radix Asteris.

Peptide are also important active ingredients in RA. 21 peptides are isolated from RA, including oligopeptides (Table 3, Figure 4 (70–72)), acyclic peptides (Table 3, Figure 4 (70–75)), and mainly chlorinated cyclic peptides (Table 3, Figure 4 (76,78–90)), among which cyclic peptides have unique anti-tumor and immunosuppressive activities [29].

### 4.4. Flavonoids

**Figure 5:**
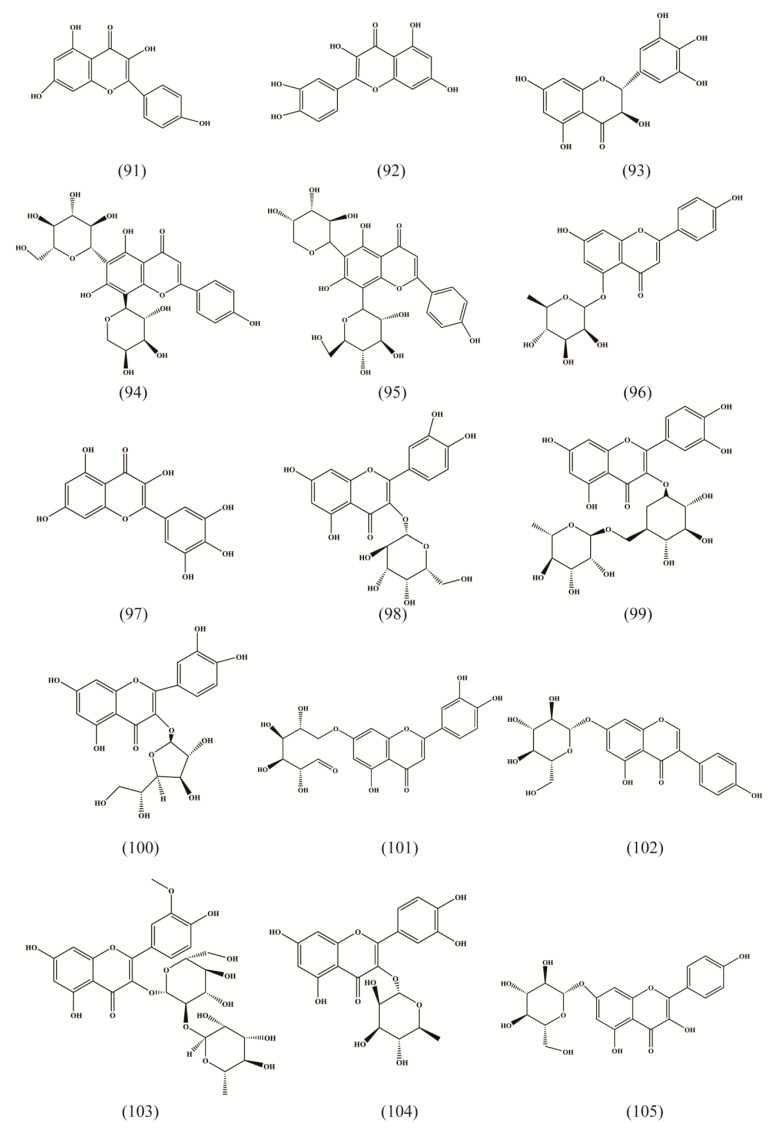

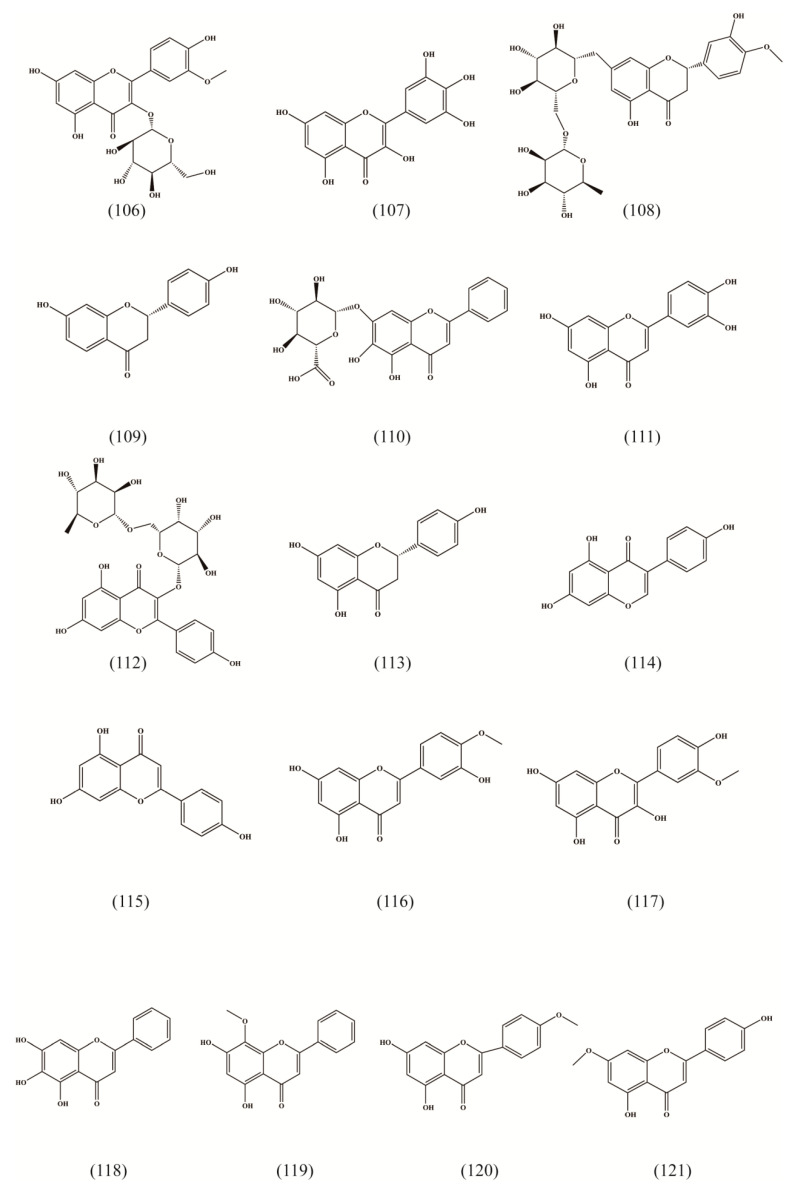
Structures of flavonoids isolated from Radix Asteris.

Flavonoids are a class of important active components in RA with multiple bioactivities, such as antioxidant and anti-hemolysis activities [30]. Two aromatic rings linked by three carbon bridges construct their basic carbon frame. A total of 32 flavonoids were found in RA, including mainly flavonoids and flavanols, as well as isoflavones (Table 3, Figure 5 (102,115)) and dihydro-flavonoids (Table 3, Figure 5 (108,109)).

### 4.5. Other Compounds

**Figure 6:**
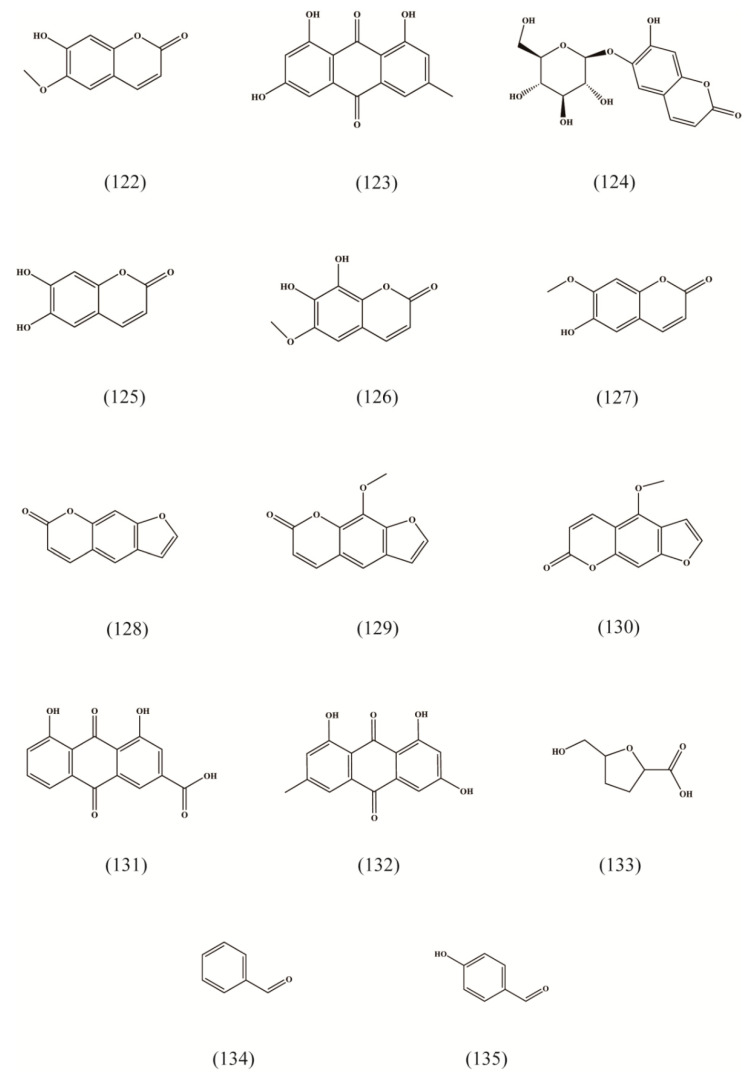
Structures of other compounds isolated from Radix Asteris.

Besides the compounds mentioned above, 14 other components are found in the roots and rhizomes of RA, such as coumarins (Table 3, Figure 6 (122,124–130)), anthraquinones (Table 3, Figure 6 (123,131,132)), and aldehydes (Table 3, Figure 6 (133–135)). Among these, emodin (Table 3, Figure 6 (132)) has a high medicinal value for its anti-tumor and anti-inflammatory activities [34].

## 5. Pharmacological Activity

**Figure 7:**
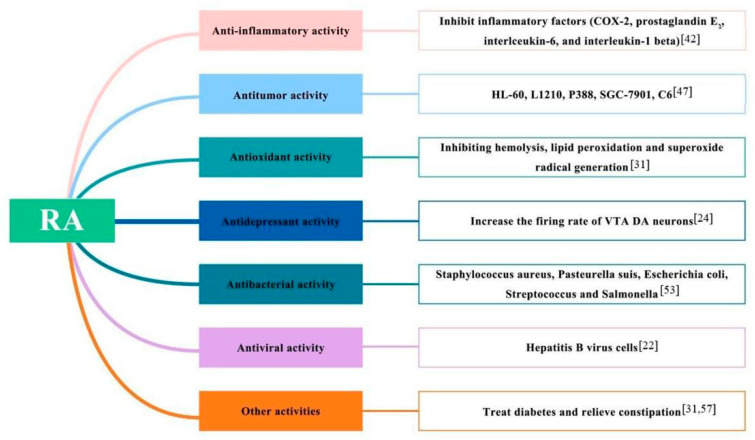
The molecular pharmacological activity mechanisms of Radix Asteri.

Numerous pharmaceutical studies of RA show its significant pharmacological activities: anti-inflammatory, antitumor, antioxidant, and antidepressant. Their molecular mechanisms are presented in Figure 7.

### 5.1. Anti-Inflammation Activity

Inflammation is a cellular response triggered by foreign stimuli and pathogen invasion and is an innate immune mechanism [35]. However, unregulated inflammation can lead to allergies, cancer and atherosclerosis [36].

Asthma is a heterogeneous disease characterized by chronic airway inflammation involving multiple cells and cellular components [37,38]. Studies have shown [39] that Fr-75 eluted in RA extract could inhibit KCl-, Ach- and KCl-, Ach- and His-induced tracheal ring contraction (3.91–250 μg/mL) possibly by reducing intracellular Ca^2+^ concentration. Therefore, it can be speculated that RA may treat asthma by inhibiting tracheal ring contraction and reducing lung inflammation.

Research by Zhang et al. [40] showed that the ethanolic extract of RA root had inhibitory effect on lipopolysaccharide (LPS)-induced C6 cell inflammation. Su et al. [6] demonstrated that aster saponin B in RA could dose-dependently suppress the inducible nitric oxide synthase (iNOS), and cyclooxygenase-2 (COX-2) protein levels were dose-dependently suppressed by aster saponin B in LPS-activated RAW 264.7 cells. Its molecular mechanism may be related to inhibition of the phosphorylation and degradation of NF-κB and subsequent prevention of the translocation of NF-κB p65 to the nucleus. Besides, lachnophyllol acetate in the root of RA could inhibit the production of inflammatory factors (Prostaglandin E2, Interleukin-6 and Interleukin-1β) and inflammatory enzymes (inducible nitric oxide synthase and cyclooxygenase 2) as a potential inhibitor for the broad treatment of inflammatory diseases [41].

**Figure 8:**
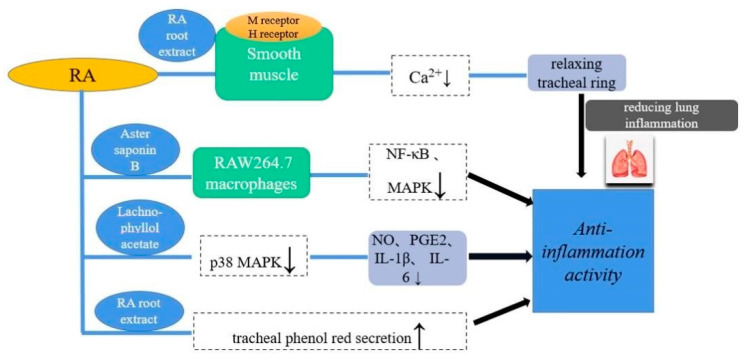
Anti-inflammatory mechanisms of Radix Asteris.

In addition, an in vivo study [3] showed that the Fr-50 fraction (40, 80 mg/kg) of a 70% ethanolic extract from RA root significantly enhanced tracheal phenol red secretion, prolonged latency, reduced cough frequency, and suppressed mouse ear edema. Wang et al. [42] found that extract of RA could reduce the edema and hemorrhage in the bladder of rats with interstitial cystitis, and extract of RA significantly reduced other pyrolysis of in vivo and in vitro death-related proteins. These results indicated that the different extracts of RA attenuated the inflammatory reaction by inhibiting various inflammatory mediators (Figure 8).

### 5.2. Anti-tumor Activity

The essence of cancer is that cells have undergone malignant changes to become malignant cells [43,44]. A variety of studies have shown that RA has a certain inhibitory effect on the growth of malignant tumor cells.

Yu et al. [17] revealed aster lingulatosides A and B from the whole plant of RA. In vitro experiments showed that they were effective against human leukemia HL-60 DNA synthesis. Cell experiments on peptides in RA carried out by Morita et al. [26] showed that cyclic peptides exhibited moderate cytotoxic activity against cultured tumor cells such as L1210 (IC50 = 15 μg/mL), P388 (IC50 = 7 μg/mL) and KB cell lines (IC50 = 14 ug/mL), exhibiting moderate cytotoxic activity, while a cyclopeptide showed no antitumor activity against S-180 ascites in vivo. Besides, the water-soluble polysaccharides isolated from RA had complete tumor growth inhibitory activity on SGC-7901 cells, indicating that polysaccharides in RA has anticancer potential [8]. This conclusion was also proved by Du Lei et al. [45] as a result of the finding that polysaccharide ATP-II in RA could inhibit the proliferation of glioma C6 cells, and lead to sustained regression of gliomas in rats and induction of apoptosis in transplanted tumor tissue. It can be seen that the inhibition of RA on tumor cells is achieved by its active components inhibiting their proliferation.

### 5.3. Antioxidation Activity

Various compounds in RA, including quercetin, kaempferol, hemoglobin and emodin, exhibited strong inhibitory effects on the generation of superoxide free radicals, in which quercetin and kaempferol could inhibit hemolysis, lipid peroxidation and superoxide radical generation [30]. Similar to quercetin and kaempferol, scopoletin and emodin also showed inhibitory effect on superoxide radical production. In addition, caffeoquinic acid in RA also has strong antioxidant effects [46,47,48].

### 5.4. Antidepressant Activity

Depression is a serious public health threat, and studies have shown that both genetic factors and mental stress can induce depression [49]. Yupeng et al. [23] used the UHPLC-Q-TOF-MS technique to identify 131 compounds in RA, and used a brain slice model to evaluate the effect of 50 of these on the ventral tegmental area (VTA). When investigating the effect of dopamine (DA) on the spontaneous firing of neurons, 5 out of 50 compounds identified in RA (i.e., chlorogenic acid, hesperidin, ferulic acid, protocatechuic acid and quercetin) were found to significantly increase the neurological effect, the effects on the firing rate of VTA DA neurons suggesting that these five compounds have significant antidepressant effects. Simultaneous determination of nine compounds in RA using HPLC-MS/MS showed that kaempferol, quercetin, chlorogenic acid, caffeic acid and ferulic acid were high, indicating that quercetin, chlorogenic acid and ferulic acid may play an important role in antidepressant [50]. However, its antidepressant mechanism still needs further research.

### 5.5. Antibacterial Activity

Xiao-Wu et al. [51] conducted an in vitro antibacterial test on the ethanol extract and alkaloid extract of RA using the test tube dilution method and the paper disc method, and the results showed that the ethanol extract of RA had strong inhibitory effects against golden yellow Staphylococcus, Pasteurella suis, Streptococcus and Salmonella. In addition, the RA alkaloid extract exhibited a strong inhibitory and antibacterial effect on Staphylococcus aureus, Pasteurella suis, Escherichia coli, Streptococcus and Salmonella.

### 5.6. Antiviral Activity

Astershionone C, a triterpenoid from the roots and rhizomes of RA, showed cytotoxic activity in B virus cells by inhibiting their DNA replication [21]. Besides, the triterpenoids shion-22-methoxy-20(21)-en-3-one and shion-22(30)-en-3,21-dione in RA exhibited inhibitory activity separately against HBeAg (IC50 = 0.83 µg/mL) and HA (IC50 = 11.18 µg/mL), as well as HBsAg (IC50 = 0.89 and 4.49 µg/mL) both [22]. At present, it is mainly reported that the Terpenoids in RA have a certain antiviral activity, but there are few studies on the antiviral principle, so further research is needed.

### 5.7. Other Activities

Besides the activities mentioned above, other bioactivities are reported. Scopoletin in RA could effectively treat diabetes and reduce oxidative stress [30]. Polyphenols rich in RA root extract could significantly reduce the body weight and blood glucose concentration of rats [46]. 

It is recorded in ancient herbal works that RA also has the effect of moisturizing the intestines and relieving constipation, which is also elucidated by modern research showing that the water decoction of RA can play a laxative role by regulating the content of neurotransmitters [52,53,54]. In an experiment in vivo, at doses of 0.16 g/mL and 0.8 g/mL, RA extract significantly promoted the transport of charcoal through the small intestine, reduced the amount of residual feces, and increased the water content of feces in the colon. In addition, RA extract could effectively relieve colon pathological damage caused by loperamide. Studies in vitro have shown that RA extract could effectively inhibit the adsorption of Ach and calcium chloride in rat duodenum. Therefore, it is speculated that RA extract may relieve constipation mainly by antagonizing the binding of acetylcholine to muscarinic receptors, inhibiting the influx of Ca^2+^ and provoking an anti-inflammatory response [55].

## 6. Toxicity

Apart from the pharmacological activities of RA, there is a lack of any systematic toxicity assessment. Only a few studies on its toxicity have been reported.

Peng et al. [56] found that different extracts (i.e., petroleum ether, ethyl acetate, n-butyl alcohol, lower aqueous phases, 75% alcohol) from RA exhibited toxicity mainly to the liver mainly, as well as the heart to a lesser extent, among which petroleum ether extract showed the strongest toxicity, followed by the ethyl acetate extract, the n-butanol extract, the low water phase extract and the 75% ethanol extract. In addition, the saponins in RA have hemolytic effects, so that preparations containing RA should not be used for intravenous injection [6].

Lei et al. [57] used serum biochemical indexes (ALT, AST, TBIL) detection and liver tissue pathological section examination to research the toxicity of RA. After a single administration, the ALT and AST serum indexes of the mice in the LD0 (0.023 g/kg) dose group were significantly increased, while there was no significant difference in TBIL index. Results of histopathological examination showed that the toxic partition of RA in the LD0 dose could cause mild cell edema in the liver of mice, and inflammatory cell infiltration and punctate necrosis of liver cells were seen in the hepatic lobules and portal areas; meanwhile, the LD100 (0.10 g/kg) dose caused different degrees of steatosis and cell edema in the mouse hepatocytes, necrosis of hepatocytes, and infiltration of inflammatory cells into the hepatic lobules and portal areas.

Cyclic peptide astin B in RA could cause oxidative stress-related inflammation in hepatocytes, as evidenced by increased reactive oxygen species (ROS) levels, decreased intracellular glutathione (GSH) levels, and enhanced c-Junn-terminal kinase (JNK) phosphorylation, as well as induction of autophagy in L-02 cells [28].

Jian-Wei et al. [58] found that oral administration of RA water decoction has strong acute toxicity causing liver injury. The LD50 dose of RA decoction could significantly increase the contents of various biochemical indexes and liver weight coefficients in serum and liver homogenates and lead to significant changes in liver tissue morphology in mice. However, the combined decoction of RA and coltsfoot can significantly reduce its toxicity, which is also one of the important reasons for the compatibility of TCM.

## 7. Quality Control

It is necessary to establish a fast, effective and accurate quality control method for TCMs due to their complexity and the diversity of their chemical components. In the 2020 edition of the Chinese Pharmacopoeia, shionone is measured by high-performance liquid chromatography (HPLC) as a marker to control the quality of RA, with a minimum total proportion of 0.15% in the “Sheng Zi Wan”, and no less than 0.10% in the “Mi Zi Wan” [1]. Currently, many measurement methods, such as thin layer chromatography, high performance liquid chromatography (HPLC), and ultra-high performance liquid chromatography (UHPLC), have been used to evaluate the quality of RA and its related products.

Kai-Xue et al. [59] measured the ointment yield and pH value of 14 batches of honey-processed RA standard decoction, and used high performance liquid chromatography to establish an HPLC fingerprint, and identify 12 common peaks, among which simple quantitative analysis of three was carried out. The evaluation method was accurate and reliable, and provided a reference for the quality control of RA and related preparations.

Gui-Mei et al. [60] used shionone as a reference substance to establish a thin-layer chromatography method to investigate 10 batches of RA. The results showed that 10 batches of honey-made RA decoction pieces showed spots with the same color in the positions corresponding to shionone, and the content of shionone was 0.12–0.24%. Therefore, this method can be used for the quality detection of honey-processed RA decoction pieces.

Guiyang et al. [61] established the HPLC fingerprint of RA medicinal materials and compared the fingerprint characteristics of eight batches of RA medicinal materials from different sources. The results showed that the method is simple, fast and accurate to detect the quality of RA.

## 8. Conclusions

This review provides a comprehensive summary of the botany, traditional uses, chemical composition, pharmacological activity, toxicity and quality control studies of RA, which is traditionally used to treat symptoms such as coughs and phlegm. Modern pharmacological studies have shown that RA has anti-inflammatory, anti-cancer, antioxidant and anti-depressant effects. Up to now, a total of 135 chemical constituents have been found in RA, among which terpenoids and flavonoids are the main components. Although many scientists have carried out a series of studies on RA, there are still shortcomings. First of all, although the research on the composition of RA is relatively comprehensive at present, RA is often used in clinical prescriptions. At present, there is a lack of research on the composition of RA, which makes it difficult for readers to know the contribution of RA to the efficacy of medicine. Second, there is a lack of a complete series of studies on RA, from animal studies to clinical studies. In addition, there are relatively few toxicological studies, and there is a lack of toxicity mechanism studies. Finally, the pharmacological research mechanism of RA and its chemical constituents mostly focuses on the regulation of signaling pathways, and lacks exploration of specific targets.

## Figures and Tables

**Table 1:** Radix Asteris prescriptions throughout the Chinese dynasties.

Title	Writer	Dynasty or Year	Characteristic and/or Indication	Dose
*Shen-Nong-Ben-Cao-Jing*	Many medical scientists in the Han Dynasty	Eastern Han Dynasty	It tastes bitter, is pungent and not toxic.	2.5–15 g
*Ming-Yi-Bie-Lu*	Hong-Jing Tao	Han Dynasty	It is pungent and non-toxic. It can treat asthma and pediatric epilepsy.	2.5–15 g
*Wu-Pu-Ben-Cao*	Pu Wu	Northern and Southern Dynasties	It is pungent and non-toxic.	2.5–15 g
*Yao-Xing-Lun*	Quan Zhen	Tang Dynasty	It is bitter in taste and flat in nature. Nourishes, treats heat deficiency	2.5–15 g
*Qian-Jin-Yi-Fang*	Si-Miao Sun	Tang Dynasty; 682 AD	It tastes bitter, is pungent and mild in nature, and is non-toxic. It can treat coughing, pus and blood, palpitations, asthma, and epilepsy in children.	2.5–15 g
*Ri-Hua-Zi-Ben-Cao*	Ri Hua Zi	Tang Dynasty	It can treat lung disease and vomiting of blood, reduce phlegm and quench thirst	2.5–15 g
*Ben-Cao-Meng-Quan*	Jia-Mo Chen	Ming Dynasty; 1565 AD	It tastes bitter and pungent, and is warm in nature. It mainly treats cough and asthma	2.5–15 g
*Ben-Cao-Gang-Mu*	Shi-Zhen Li	Ming Dynasty; 1578 AD	It tastes bitter, warm in nature, non-toxic, and mainly treats coughs	2.5–15 g
*Jing-Yue-Quan-Shu*	Jie-Bin Zhang	Ming Dynasty; 1624 AD	It is bitter and pungent, treats cough and asthma	2.5–15 g
*Ben-Cao-Dong-Quan*	Mu Shen	Qing Dynasty; 1661 AD	It tastes bitter, and is warm in nature, non-toxic, it regulates the spleen and stomach, relieves phlegm and relieves cough	2.5–15 g
*Ben-Cao-Xiang-Jie*	Yue Min	Qing Dynasty; 1681 AD	It tastes bitter and pungent, and mainly treats blood phlegm	2.5–15 g
*Ben-Cao-Bei-Yao*	Ang Wang	Qing Dynasty; 1694 AD	It is pungent and warm in nature, can nourish the lungs, mainly treats cough and blood in sputum	2.5–15 g
*Ben-Cao-Bian-Du*	Bing-Cheng Zhang	Qing Dynasty; 1887 AD	It is warm in nature and treats wind-cold cough	2.5–15 g

**Table 2:** Traditional prescriptions containing Radix Asteris.

Preparation Name	Composition	Preparations	Route of Administration	Dosing Frequency	Clinic Use	Reference
Shegan Mahuang Soup	*Belamcanda chinensis*(L.) DC., *Ephedra sinica* Stapf, *Zingiber officinale* Rosc., *Asarum sieboldii* Miq., *Aster tataricus* L. f., *Tussilago farfara* L., *Schisandra chinensis* (Turcz.) Baill., *Ziziphus jujuba* Mill., *Pinellia ternata* (Thunb.) breit.	Decoction	Oral administration	b.i.d	cold phlegm stagnation lung and throat syndrome	*Jin-Gui-Yao-Lve*, Han Dynasty
Ze Qi Soup	*Pinellia ternata* (Thunb.) Breit., *Aster tataricus* L. f., *Euphorbia helioscopia* L., *Zingiber officinale* Rosc., *Cynanchum* *glaucescens* (Decne.) Hand.-Mazz., *Glycyrrhiza uralensis* Fisch., *Scutellaria baicalensis* Georgi, *Panax ginseng* C. A. Mey., *Cinnamomum cassia* Presl	Decoction	Oral administration	b.i.d	Occasional wheezing and coughing, body swelling, restlessness	*Jin-Gui-Yao-Lve*, Han Dynasty
Zi Wan Soup	*Glycyrrhiza uralensis* Fisch., *Aster tataricus* L. f., *Morus alba* L., *Platycodon grandiflorum* (Jacq.) A.DC., *Prunus armeniaca* L.var.ansu Maxim., *Asparagus cochinchinensis* (Lour.) Merr., *Bambusa tuldoides* Munro	Decoction	Oral administration	b.i.d	Obstructed throat, shortness of breath	*Sheng-Ji-Zong-Lu*, Han Dynasty
Bai Bu Powder	*Stemona sessilifolia* (Miq.) Miq., *Fritilaria cirrhosa* D.Don, *Aster tataricus* L. f., *Pueraria lobata* (Willd.) Ohwi, Gypsum	Decoction	Oral administration	b.i.d	Cough and fever in children	*Tai-Ping-Sheng-Hui-Fang*, Song Dynasty
Zi Wan Pills	*Aster tataricus* L. f., *Rubia cordifolia* L.	Pill	Oral administration	b.i.d	Cough, hematemesis, hemoptysis due to lung injury	*Ji-Feng-Pu-Ji-Fang*, Song Dynasty
Zi Wan Bai Hua Powder	*Aster tataricus* L. f., *Tussilago farfara* L., *Stemona sessilifolia* (Miq.) Miq.	Decoction	Oral administration	b.i.d	persistent cough	*Tu-Jing-Ben-Cao*, Song Dynasty
Luo Shi Soup	*Trachelos permum jasminoides* (Lindl.) Lem., *Aster tataricus* L. f., *Cimicifuga foetida* L., *Belamcanda chinensis* (L.) DC., *Platycodon grandiflorum* (Jacq.) A.DC., *Akebia quinata* (Thunb.) Decne, *Poria cocos* (Schw.) Wolf.	Decoction	Oral administration	b.i.d	Choking in the throat	*Zheng-He-Sheng-Ji-Zong-Lu*, Yuan Dynasty
Bu Fei Soup	*Panax ginseng* C. A. Mey., *Astragalus membranaceus* (Fisch.) Bge., *Rehmannia glutinosa* Libosch., *Schisandra chinensis* (Turcz.) Baill., *Aster tataricus* L. f., *Morus alba* L.	Decoction	Oral administration	b.i.d	Lung deficiency cough and asthma	*Yong-Lei-Qian-Fang*, Yuan Dynasty
Zhi Sou Powder	*Platycodon grandiflorum* (Jacq.) A.DC., *Schizonepeta tenuifolia* Briq., *Aster tataricus* L. f., *Stemona sessilifolia* (Miq.) Miq., *Cynanchum* *glaucescens* (Decne.) Hand.-Mazz., *Glycyrrhiza uralensis* Fisch., *Citrus reticulata* Blanco	Powder	Oral administration	b.i.d	acute and chronic bronchitis	*Yi-Xue-Xin-Wu*, Qing Dynasty
Er Zi Soup	*Perilla frutescens* (L.) Britt., *Aster tataricus* L. f., *Platycodon grandiflorum* (Jacq.) A.DC., *Glycyrrhiza uralensis* Fisch., *Citrus aurantium* L., *Scutellaria baicalensis* Georgi., *Trichosanthes kirilowii* Maxim.	Decoction	Oral administration	b.i.d	stuffy nose and cough	*Bian-Zheng-Lu*, Qing Dynasty

**Table 3:** Compounds and activities isolated from Radix Asteris.

No	Compound Name	Resource	References
Terpenes			
1	Shionoside A	Roots	T. Nagao et al. [10]
2	Shionoside B	Roots	T. Nagao et al. [10]
3	Epifriedelinol	Roots	T. Nagao et al. [10]
4	Aster saponin A	Roots	T. Nagao et al. [11]
5	Aster saponin B	Roots	T. Nagao et al. [11]
6	Aster saponin C	Roots.	T. Nagao et al. [11]
7	Aster saponin D	Roots	T. Nagao et al. [11]
8	Aster saponin E	Roots	T. Nagao et al. [12]
9	Aster saponin F	Roots	T. Nagao et al. [12]
10	Aster saponin Ha	The ground part	T. Nagao et al. [13]
11	Aster saponin Hb	The ground part	T. Nagao et al. [13]
12	Aster saponin Hc	The ground part	T. Nagao et al. [13]
13	Aster saponin Hd	The ground part	T. Nagao et al. [13]
14	Foetidissimoside A	The ground part	T. Nagao et al. [13]
15	Aster batanoside F	Roots	Y. Shao et al. [14]
16	Aster batanoside B	Roots	Y. Shao et al. [15]
17	Aster batanoside C	Roots	Y. Shao et al. [15]
18	Aster lingulatoside A	The whole plants	S. Yu et al. [16]
19	Aster lingulatoside B	The whole plants	S. Yu et al. [16]
20	Aster lingulatoside C	The whole plants	Y. Shao et al. [17]
21	Aster lingulatoside D	The whole plants	Y. Shao et al. [17]
22	Astertarone A	Roots	Akihisa et al. [18]
23	Shionone	Roots and rhizomes	Akihisa et al. [18]
24	Friedelin	Roots and rhizomes	Akihisa et al. [18]
25	Astertarone B	Roots	A. Toshihiro et al. [19]
26	Friedelan-3-ol	Roots	V. Lanzotti et al. [20]
27	Aster shionone A	Roots and rhizomes	W.B. Zhou et al. [21]
28	Aster shionone B	Roots and rhizomes	W.B. Zhou et al. [21]
29	Aster shionone C	Roots and rhizomes	W.B. Zhou et al. [21]
30	Aster shionone D	Roots and rhizomes	W.B. Zhou et al. [21]
31	Aster shionone E	Roots and rhizomes	W.B. Zhou et al. [21]
32	Aster shionone F	Roots and rhizomes	W.B. Zhou et al. [21]
33	Shion-22(30)-en-3,21-dione	Rhizomes	B.Z. Wen et al. [22]
34	Shion-22-methoxy-20(21)-en-3-one	Rhizomes	B.Z. Wen et al. [22]
35	Shion-22-methoxy-20(21)-en-3β-ol	Rhizomes	B.Z. Wen et al. [22]
36	2,3,24-Trihydroxyolean-12-en-28-oic acid	Roots and rhizomes	S. Yupeng et al. [23]
37	23-Hydroxybetulinic acid	Roots and rhizomes	S. Yupeng et al. [23]
38	Echinocystic acid	Roots and rhizomes	S. Yupeng et al. [23]
39	Betulinic acid	Roots and rhizomes	S. Yupeng et al. [23]
40	Oleanic acid	Roots and rhizomes	S. Yupeng et al. [23]
41	Taraxerol	Roots and rhizomes	S. Yupeng et al. [23]
42	Betulin	Roots and rhizomes	S. Yupeng et al. [23]
43	Taraxasterol	Roots and rhizomes	S. Yupeng et al. [23]
44	Beta-Amyrin	Roots and rhizomes	S. Yupeng et al. [23]
45	3-O-α-L-arabinopyranosyl- (1→6)-β-D-trihydroxyolean-12-en-28-oic acid	The underground parts	X.D. Su et al. [6]
46	Aster saponin G	The underground parts	X.D. Su et al. [6]
47	Aster saponin C2	The underground parts	X.D. Su et al. [6]
48	Aster saponin A2	The underground parts	X.D. Su et al. [6]
49	Aster saponin G2	The underground parts	X.D. Su et al. [6]
50	Aster saponin H	The underground parts	X.D. Su et al. [6]
Organic acids			
51	Pyrogallic acid	Roots and rhizomes	S. Yupeng et al. [23]
52	Protocatechuate	Roots and rhizomes	S. Yupeng et al. [23]
53	Chlorogenic acid	Roots and rhizomes	S. Yupeng et al. [23]
54	Caffeic acid	Roots and rhizomes	S. Yupeng et al. [23]
55	Ferulic acid	Roots and rhizomes	S. Yupeng et al. [23]
56	Benzoic acid	Roots and rhizomes	S. Yupeng et al. [23]
57	Isoferulic acid	Roots and rhizomes	S. Yupeng et al. [23]
58	Methyl caffeate	Roots and rhizomes	S. Yupeng et al. [23]
59	Cynarin	Roots and rhizomes	S. Yupeng et al. [23]
60	Paeonol	Roots and rhizomes	S. Yupeng et al. [23]
61	Succinic acid	Roots and rhizomes	S. Yupeng et al. [23]
62	2,2-dimethylsuccinic acid	Roots and rhizomes	S. Yupeng et al. [23]
63	4-hydroxybenzoic acid	Roots and rhizomes	S. Yupeng et al. [23]
64	Cryptochlorogenic acid	Roots and rhizomes	S. Yupeng et al. [23]
65	3,4-dicaffeoylquinic acid	Roots and rhizomes	S. Yupeng et al. [23]
66	3,5-dicaffeoylquinic acid	Roots and rhizomes	S. Yupeng et al. [23]
67	4,5-dicaffeoylquinic acid	Roots and rhizomes	S. Yupeng et al. [23]
68	Docosyl caffeate separately	Roots and rhizomes	S. Yupeng et al. [23]
69	Vanillic acid	Roots and rhizomes	S. Yupeng et al. [23]
Peptides			
70	Asterinin A	Roots	D. Cheng et al. [24]
71	Asterinin B	Roots	D. Cheng et al. [24]
72	Asterinin C	Roots	D. Cheng et al. [24]
73	Astin J	Roots	H. Morita et al. [25]
74	Asterinin D	Roots	D.L. Cheng et al. [26]
75	Asterinin E	Roots	D.L. Cheng et al. [26]
76	Astin H	Roots	H. Morita et al. [27]
77	Astin G	Roots	H. Morita et al. [27]
78	Astin E	Roots	H. Morita et al. [27]
79	Astin I	Roots	H. Morita et al. [27]
80	Astin F	Roots	H. Morita et al. [27]
81	Astin D	Roots	H. Morita et al. [27]
82	Astin A	Roots	H. Morita et al. [27]
83	Astin B	Roots	H. Morita et al. [27,28]
84	Astin C	Roots	H. Morita et al. [27]
85	Astin K	Roots and rhizomes	H. Xu et al. [29]
86	Astin M	Roots and rhizomes	H. Xu et al. [29]
87	Astin N	Roots and rhizomes	H. Xu et al. [29]
88	Astin O	Roots and rhizomes	H. Xu et al. [29]
89	Astin P	Roots and rhizomes	H. Xu et al. [29]
90	Astin L	Roots and rhizomes	S. Yupeng et al. [23]
Flavonoids			
91	Kaempferol	Roots and rhizomes	T.B. Ng et al. [30]
92	Quercetin	Roots and rhizomes	S. Yupeng et al. [23,30]
93	Dihydromyricetin	Roots and rhizomes	S. Yupeng et al. [23]
94	Schaftoside	Roots and rhizomes	S. Yupeng et al. [23]
95	Isoschaftoside	Roots and rhizomes	S. Yupeng et al. [23]
96	Apigenin-5- rhamnoside	Roots and rhizomes	S. Yupeng et al. [23]
97	Myrictrin	Roots and rhizomes	S. Yupeng et al. [23]
98	Hyperoside	Roots and rhizomes	S. Yupeng et al. [23]
99	Rutin	Roots and rhizomes	S. Yupeng et al. [23]
100	Isoquercitrin	Roots and rhizomes	S. Yupeng et al. [23]
101	Luteolin-7- galacturonide	Roots and rhizomes	S. Yupeng et al. [23]
102	Genistin	Roots and rhizomes	S. Yupeng et al. [23]
103	Isorhamnetin-3-O- neohespeidoside	Roots and rhizomes	S. Yupeng et al. [23]
104	Quercitrin	Roots and rhizomes	S. Yupeng et al. [23]
105	Kaempferol-7-O-β-D-glucopyranoside	Roots and rhizomes	S. Yupeng et al. [23]
106	Isorhamnetin-3-O- glucoside	Roots and rhizomes	S. Yupeng et al. [23]
107	Myricetin	Roots and rhizomes	S. Yupeng et al. [23]
108	Hesperidin	Roots and rhizomes	S. Yupeng et al. [23]
109	Liquiritigenin	Roots and rhizomes	S. Yupeng et al. [23]
110	Baicalin	Roots and rhizomes	S. Yupeng et al. [23]
111	Luteolin	Roots and rhizomes	S. Yupeng et al. [23]
112	Biorobin	Roots and rhizomes	S. Yupeng et al. [23]
113	Naringenin	Roots and rhizomes	S. Yupeng et al. [23]
114	Genistein	Roots and rhizomes	S. Yupeng et al. [23]
115	Apigenin	Roots and rhizomes	S. Yupeng et al. [23]
116	Diosmetin	Roots and rhizomes	S. Yupeng et al. [23]
117	Isorhamnetin	Roots and rhizomes	S. Yupeng et al. [23]
118	Baicalein	Roots and rhizomes	S. Yupeng et al. [23]
119	Wogonin	Roots and rhizomes	S. Yupeng et al. [23]
120	Acacetin	Roots and rhizomes	S. Yupeng et al. [23]
121	Genkwanin	Roots and rhizomes	S. Yupeng et al. [23]
Other compounds			
122	Scopoletin	Roots and rhizomes	T.B. Ng et al. [30]
123	Emodin	Roots and rhizomes	T.B. Ng et al. [30]
124	Esculin	Roots and rhizomes	S. Yupeng et al. [23]
125	Esculetin	Roots and rhizomes	S. Yupeng et al. [23]
126	Fraxetin	Roots and rhizomes	S. Yupeng et al. [23]
127	Isoscopoletin	Roots and rhizomes	S. Yupeng et al. [23]
128	Psoralen	Roots and rhizomes	S. Yupeng et al. [23]
129	Xanthotoxin	Roots and rhizomes	S. Yupeng et al. [23]
130	Bergapten	Roots and rhizomes	S. Yupeng et al. [23]
131	Rhein	Roots and rhizomes	S. Yupeng et al. [23]
132	Emodin anthrone	Roots and rhizomes	S. Yupeng et al. [23]
133	5-Hydroxymethyl-2- furaldehyde	Roots and rhizomes	S. Yupeng et al. [23]
134	Benzaldehyde	Roots and rhizomes	S. Yupeng et al. [23]
135	p-Hydroxybenzaldehyde	Roots and rhizomes	S. Yupeng et al. [23]

## References

[1] Chinese Pharmacopoeia Commission (2020). Pharmacopoeia of the People’s Republic of China.

[2] Editorial Board of Flora of China (Ed.) (1992). Flora of China.

[3] Yu P., Cheng S., Xiang J., Yu B., Zhang M., Zhang C., Xu X. (2015). Expectorant, antitussive, anti-inflammatory activities and compositional analysis of *Aster tataricus*. J. Ethnopharmacol..

[4] Jiang K., Song Q., Wang L., Xie T., Wu X., Wang P., Yin G., Ye W., Wang T. (2014). Antitussive, expectorant and anti-inflammatory activities of different extracts from Exocarpium *Citri grandis*. J. Ethnopharmacol..

[5] Liu K.Y., Zhang T.J., Gao W.Y., Zheng Y.N., Chen H.X. (2006). Triterpenes and Steroids from *Aster tataricus*. Nat. Prod. Res..

[6] Su X.D., Jang H.J., Wang C.Y., Lee S.W., Rho M.C., Kim Y.H., Yang S.Y. (2019). Anti-inflammatory Potential of Saponins from *Aster tataricus* via NF-kappaB/MAPK Activation. J. Nat. Prod..

[7] Liu Z., Xi R., Zhang Z., Li W., Liu Y., Jin F., Wang X. (2014). 4-Hydroxyphenylacetic acid attenuated inflammation and edema via suppressing HIF-1alpha in seawater aspiration-induced lung injury in rats. Int. J. Mol. Sci..

[8] Zhang Y., Wang Q., Wang T., Zhang H., Tian Y., Luo H., Yang S., Wang Y., Huang X. (2012). Inhibition of human gastric carcinoma cell growth in vitro by a polysaccharide from *Aster tataricus*. Int. J. Biol. Macromol..

[9] Xiu Y.F., Cheng X.M., Liu L., Wu T., Wang Z.T. (2006). Comparison of shionone content in different slices of prepared radix asteris. J. Shanghai Univ. TCM.

[10] Nagao T., Okabe H., Yamauchi T. (1988). Studies on the Constituents of *Aster tataricus* L. f. I.: Structures of Shionosides a and B: Monoterpene Glycosides Isolated from the Root. Chem. Pharm. Bull..

[11] Nagao T., Hachiyama S., Okabe H., Yamauchi T. (1989). Studies on the Constituents of *Aster tataricus* L. f. II: Structures of Aster Saponins Isolated from the Root. Chem. Pharm. Bull..

[12] Nagao T., Okabe H., Yamauchi T. (1990). Studies on the Constituents of *Aster tataricus* L. f. III: Structures of Aster Saponins E and F Isolated from the Root. Chem. Pharm. Bull..

[13] Tanaka R., Nagao T., Okabe H., Yamauchi T. (1990). Studies on the Constituents of *Aster tataricus* L. f. IV: Structures of Aster Saponins Isolated from the Herb. Chem. Pharm. Bull..

[14] Shao Y., Zhou B.N., Lin L.Z., Cordell G.A. (1995). Triterpenoid saponins from *Aster batangensis*. Phytochemistry.

[15] Shao Y., Li Y.L., Zhou B.N. (1996). Phenolic and triterpenoid glycosides from *Aster batangensis*. Phytochemistry.

[16] Yu S., Chi-Tang H., Chee-Kok C., Robert T.R., Bin H., Guo-Wei Q. (1997). Triterpenoid saponins from *Aster lingulatus*. Phytochemistry.

[17] Shao Y., Ho C., Chin C., Poobrasert O., Yang S., Cordell G.A. (1997). Asterlingulatosides C and D, Cytotoxic Triterpenoid Saponins from *Aster lingulatus*. J. Nat. Prod..

[18] Akihisa T., Kimura Y., Koike K., Yasukawa K., Arai K., Suzuki Y., Nikaido T. (1998). Astertarone A: A Triterpenoid Ketone Isolated from the Roots of *Aster tataricus* L.. Chem. Pharm. Bull..

[19] Toshihiro A., Yumiko K., Takaaki T., Koichi A. (1999). Astertarone B, a Hydroxy-Triterpenoid Ketone from the Roots of *Aster tataricus* L.. Chem. Pharm. Bull..

[20] Lanzotti V. (2005). Bioactive Saponins from Allium and Aster Plants. Phytochem. Rev..

[21] Zhou W.B., Zeng G.Z., Xu H.M., He W.J., Zhang Y.M., Tan N.H. (2014). Astershionones A-F, six new anti-HBV shionane-type triterpenes from *Aster tataricus*. Fitoterapia.

[22] Wen B.Z., Jun Y.T., Hui M.X., Ke L.C., Guang Z.Z., Chang J.J., Yu M.Z., Ning H.T. (2014). Three New Antiviral Triterpenes from *Aster tataricus*. Z. Naturforsch..

[23] Yupeng S., Li L., Man L., Min S., Changchen W., Lantong Z., Hailin Z. (2018). A systematic data acquisition and mining strategy for chemical profiling of *Aster tataricus* rhizoma (Ziwan) by UHPLC-Q-TOF-MS and the corresponding anti-depressive activity screening. J. Pharmaceut. Biomed..

[24] Cheng D., Shao Y., Hartman R., Roder E., Zhao K. (1994). Oligopeptides from *Aster tataricus*. Phytochemistry.

[25] Morita H., Nagashima S., Takeya K., Itokawa H. (1995). Structure of a new peptide, astin J, from *Aster tataricus*. Chem. Pharm. Bull..

[26] Cheng D.L., Shao Y., Zhao K., Hartmann R., Roeder E. (1996). Pentapeptides from the roots of *Aster tataricus*. Pharmazie.

[27] Morita H., Nagashima S., Uchiumi Y., Kuroki O., Takeya K., Itokawa H. (1996). Cyclic peptides from higher plants. XXVIII. Antitumor activity and hepatic microsomal biotransformation of cyclic pentapeptides, astins, from *Aster tataricus*. Chem. Pharm. Bull..

[28] Wang L., Li M.D., Cao P.P., Zhang C.F., Huang F., Xu X.H., Liu B.L., Zhang M. (2014). Astin B, a cyclic pentapeptide from *Aster tataricus*, induces apoptosis and autophagy in human hepatic L-02 cells. Chem. Biol. Interact..

[29] Xu H., Zeng G., Zhou W., He W., Tan N. (2013). Astins K–P, six new chlorinated cyclopentapeptides from *Aster tataricus*. Tetrahedron..

[30] Ng T.B., Liu F., Lu Y., Cheng C.H., Wang Z. (2003). Antioxidant activity of compounds from the medicinal herb *Aster tataricus*. Comp. Biochem. Physiol. C Toxicol Pharmacol..

[31] Campbell J.B., Peerbaye Y.A. (1992). Saponin. Res. Immunol..

[32] Zhao D., Hu B., Zhang M., Zhang C., Xu X. (2015). Simultaneous separation and determination of phenolic acids, pentapeptides, and triterpenoid saponins in the root of *Aster tataricus* by high-performance liquid chromatography coupled with electrospray ionization quadrupole time-of-flight mass spectrometry. J. Sep. Sci..

[33] Yang H., Shi H., Zhang Q., Liu Y., Wan C., Zhang L. (2016). Simultaneous determination of five components in *Aster tataricus* by ultra performance liquid chromatography–tandem mass spectrometry. J. Chromatogr. Sci..

[34] Dong X., Fu J., Yin X., Cao S., Li X., Lin L., Ni J. (2016). Emodin: A review of its pharmacology, toxicity and pharmacokinetics. Phytother. Res..

[35] Barton G.M. (2008). A calculated response: Control of inflammation by the innate immune system. J.Clin. Investig..

[36] Ferrero-Miliani L., Nielsen O.H., Andersen P.S., Girardin S.E. (2007). Chronic inflammation: Importance of NOD2 and NALP3 in interleukin-1beta generation. Clin. Exp. Immunol..

[37] Bates J.H., Rincon M., Irvin C.G. (2009). Animal models of asthma. Am. J. Physiol. Lung Cell Mol. Physiol..

[38] Lee H.Y., Kim I.K., Yoon H.K., Kwon S.S., Rhee C.K., Lee S.Y. (2017). Inhibitory effects of resveratrol on airway remodeling by transforming growth Factor-beta/Smad signaling pathway in chronic asthma model. Allergy Asthma Immunol. Res..

[39] Chen Y., Wu H., Li Y., Liu J., Jia Z., Xu W., Xiao H., Wang W. (2020). *Aster tataricus* attenuates asthma efficiently by simultaneously inhibiting tracheal ring contraction and inflammation. Biomed. Pharmacother..

[40] Zhang H., Tian M., He Q., Chi N., Xiu C., Wang Y. (2017). Effect of *Aster tataricus* on production of inflammatory mediators in LPS stimulated rat astrocytoma cell line (C6) and THP-1 cells. Saudi Pharm. J..

[41] Xiang D.S., Hyun-Jae J., Hong X.L., Young H.K., Seo Y.Y. (2019). Identification of potential inflammatory inhibitors from *Aster tataricus*. Bioorg. Chem..

[42] Wang X., Fan L., Yin H., Zhou Y., Tang X., Fei X., Tang H., Peng J., Ren X., Xue Y. (2020). Protective effect of *Aster tataricus* extract on NLRP3-mediated pyroptosis of bladder urothelial cells. J. Cell. Mol. Med..

[43] Ohgaki H., Kleihues P. (2005). Epidemiology and etiology of gliomas. Acta. Neuropathol..

[44] Fisher J.L., Schwartzbaum J.A., Wrensch M., Wiemels J.L. (2007). Epidemiology of brain tumors. Neurol. Clin..

[45] Du L., Mei H.F., Yin X., Xing Y.Q. (2014). Delayed growth of glioma by a polysaccharide from *Aster tataricus* involve upregulation of Bax/Bcl-2 ratio, activation of caspase-3/8/9, and downregulation of the Akt. Tumour Biol..

[46] Du H., Zhang M., Yao K., Hu Z. (2017). Protective effect of Aster tataricus extract on retinal damage on the virtue of its antioxidant and anti-inflammatory effect in diabetic rat. Biomed. Pharmacother..

[47] Ma C., Dastmalchi K., Whitaker B.D., Kennelly E.J. (2011). Two new antioxidant malonated caffeoylquinic acid isomers in fruits of wild eggplant relatives. J. Agric. Food Chem..

[48] Peluso G., de Feo V., de Simone F., Bresciano E., Vuotto M.L. (1995). Studies on the inhibitory effects of caffeoylquinic acids on monocyte migration and superoxide ion production. J. Nat. Prod..

[49] de Oliveira M.R., Chenet A.L., Duarte A.R., Scaini G., Quevedo J. (2018). Molecular mechanisms underlying the anti-depressant effects of resveratrol: A review. Mol. Neurobiol..

[50] Wan C.C., Liu Y.Y., Yang H.T., Zhang Q.Y., Liao M., Zhang X., Zhang L.T. (2016). Simultaneous determination of nine constituents in Asteris Radix by HPLC-MS/MS. Chin. Tradit. Herb. Drugs..

[51] Xiao-Wu T., Xiang-Xin L., Yu-Long T., Ya-Lin L., Kang-Hui X. (2016). Analysis of effective constituents from *Aster tataricus* L. F. And extracting of alkaloid and its antibacterial test in vitro. J. Tradit. Chin. Vet. Med..

[52] Jia Z.X., Zhi K.Y., Li J.L. (2012). Small talk about *Aster Tataricus*. Shanxi J. Tradit. Med. Chin..

[53] Jia Z.X., Wang Y.H., Feng W.J., Zhi K.Y., Gong L. (2011). Experience research on the defecated function of *Aster Tataricus*. Guangming J. Chin. Med..

[54] Li Y., Liang H. (2017). Investigation on effects of *Aster tataricus* on nourishing kidney and promoting urination and defecation. Shanghai J. Tradit. Chin. Med..

[55] Wu H., Chen Y., Huang B., Yu Y., Zhao S., Liu J., Jia Z., Xiao H. (2021). *Aster tataricus* alleviates constipation by antagonizing the binding of acetylcholine to muscarinic receptor and inhibiting Ca(2+) influx. Biomed. Pharmacother..

[56] Peng W.J., Xin R.H., Luo Y.J., Liang G., Ren L.H., Liu Y., Wang G.B., Zheng J.F. (2016). Evaluation of the acute and subchronic toxicity of *Aster tataricus* L.F. Afr. J Tradit. Complement Altern. Med..

[57] Lei W., Mian Z., Jing J., Fang H., Chao-Feng Z. (2010). Toxic fraction of radix asteris and its acute hepatotoxicity to mice. Lishizhen Med. Mater. Med. Res..

[58] Zhang J.W., Dou C.G., Zhang M., Ma S.P., Huang F. (2007). Toxicity of Radix Asteris, Flos Farfarae and their combination. Chin. J. Clin. Pharm. Ther..

[59] Xue Z.K., Wei M., Chao O., Weili S., Wen X., Lan W. (2018). Preparation and quality evaluation of the standard decoction of asteris radix et rhizoma praeparata cum melle. J. Chin. Med. Mater..

[60] Gui-Mei L., You-Xue L., Zi-Xiao Z., Tian-Zhu J. (2017). Optimization of quality standard of honey preparation process of Radix Asteris. Lishizhen Med. Mater. Med. Res..

[61] Guiyang C., Rui Z., Jinhai H. (2015). Study on HPLC Fingerprint of Aster Medicinal Materials. Heilongjiang J. TCM.

